# Hallucination and Anæsthetics

**Published:** 1896-12

**Authors:** 


### Hallucination and Anæsthetics.

In the case of State vs. Perry, the Supreme Court of Appeals of West Virginia held that expert medical testimony should determine whether or not hallucination, while under the influence of an anæsthetic, was responsible for criminal charges preferred against a physician by a female patient. If the probability of such hallucination is established and the charges rest entirely on uncorroborated testimony of the patient, the jury, it held, should acquit the accused.

